# Removal of Chromophore-Proximal Polar Atoms Decreases Water Content and Increases Fluorescence in a Near Infrared Phytofluor

**DOI:** 10.3389/fmolb.2015.00065

**Published:** 2015-11-25

**Authors:** Heli Lehtivuori, Shyamosree Bhattacharya, Nicolaas M. Angenent-Mari, Kenneth A. Satyshur, Katrina T. Forest

**Affiliations:** ^1^Department of Bacteriology, University of Wisconsin-MadisonMadison, WI, USA; ^2^Department of Physics, Nanoscience Center, University of JyväskyläJyväskylä, Finland

**Keywords:** chromophore binding domain (CBD), *Deinococcus radiodurans*, Wisconsin infrared phytofluor (WiPhy2), tetrapyrrole, excitation-emission matrix (EEM)

## Abstract

Genetically encoded fluorescent markers have revolutionized cell and molecular biology due to their biological compatibility, controllable spatiotemporal expression, and photostability. To achieve *in vivo* imaging in whole animals, longer excitation wavelength probes are needed due to the superior ability of near infrared light to penetrate tissues unimpeded by absorbance from biomolecules or autofluorescence of water. Derived from near infrared-absorbing bacteriophytochromes, phytofluors are engineered to fluoresce in this region of the electromagnetic spectrum, although high quantum yield remains an elusive goal. An invariant aspartate residue is of utmost importance for photoconversion in native phytochromes, presumably due to the proximity of its backbone carbonyl to the pyrrole ring nitrogens of the biliverdin (BV) chromophore as well as the size and charge of the side chain. We hypothesized that the polar interaction network formed by the charged side chain may contribute to the decay of the excited state via proton transfer. Thus, we chose to further probe the role of this amino acid by removing all possibility for polar interactions with its carboxylate side chain by incorporating leucine instead. The resultant fluorescent protein, WiPhy2, maintains BV binding, monomeric status, and long maximum excitation wavelength while minimizing undesirable protoporphyrin IXα binding in cells. A crystal structure and time-resolved fluorescence spectroscopy reveal that water near the BV chromophore is excluded and thus validate our hypothesis that removal of polar interactions leads to enhanced fluorescence by increasing the lifetime of the excited state. This new phytofluor maintains its fluorescent properties over a broad pH range and does not suffer from photobleaching. WiPhy2 achieves the best compromise to date between high fluorescence quantum yield and long illumination wavelength in this class of fluorescent proteins.

## Introduction

Fluorophores active in the near infrared (NIR) attract ongoing attention due to their diverse applications in biomedical research, materials science and related fields. They allow imaging with minimal autofluorescence and light scattering in animals, and deep tissue penetration (Weissleder, 2001). *In vivo*, real-time advanced imaging studies and vascular mapping of the heart and brain, the visualization of tumors and plaques, and guided surgery are aspects of fundamental research and translational applications that will benefit substantially from the creation of improved NIR fluorophores. The development of simple, stable, non-toxic, modular, and small molecular weight NIR platforms is thus of great interest to the biomedical community and has proceeded both in the realm of chemical biology and fluorescent proteins. In the former category, there are several classes of small molecule NIR dyes available including nanoparticles, cyanine dyes, phthalocyanine, and squaraine dyes (Escobedo et al., 2010; Hahn et al., 2011; Luo et al., 2011; Gibbs, 2012; Battistelli et al., 2015). The promise of genetically encoded NIR fluorescent proteins has, on the other hand, led to a renaissance in research of engineered fluorescent proteins, based both on Green Fluorescent Protein-like β-barrel folds and more recently on bacteriophytochromes (Zhang et al., 2002; Gibbs, 2012; Guo et al., 2014; Marx, 2014).

**Figure 1:**
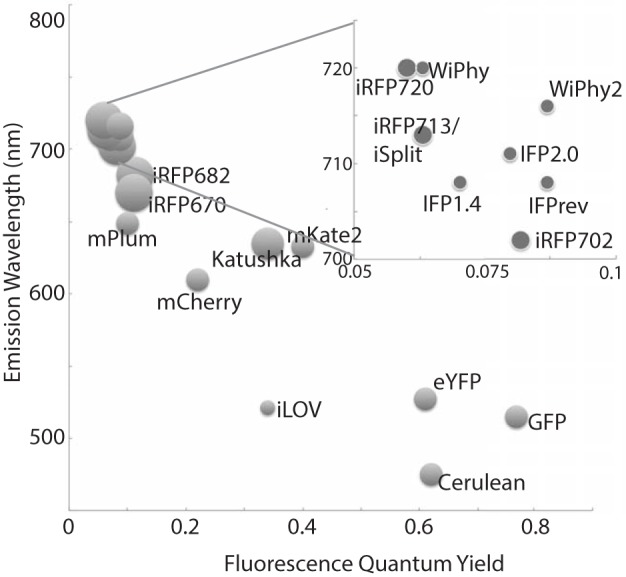
**A representative sampling of historical and currently favored genetically-encoded fluorescent probes are classified by excitation wavelength and fluorescence quantum yield**. The relative size of each fluorophore, taking both polypeptide molecular mass and oligomeric status into account, is proportional to the diameter of its marker. Quantum yields and emission wavelengths are taken from the literature (Johnson et al., 1962; Ormö et al., 1996; Rizzo et al., 2004; Shaner et al., 2004; Shcherbo et al., 2007, 2009; Shu et al., 2009; Auldridge et al., 2012; Christie et al., 2012; Filonov and Verkhusha, 2013; Shcherbakova and Verkhusha, 2013; Yu et al., 2014) except for WiPhy2 (this work).

Bacteriophytochromes (BphPs) are promising design templates for NIR fluorescent proteins. Their covalent association with the linear tetrapyrrole biliverdin IXα (BV) allows BphPs to absorb light in the red and far-red region of the spectrum. As an intermediate of normal mammalian heme catabolism, BV does not necessarily need to be provided exogenously in order to achieve *in vivo* fluorescence. Thus, great effort has gone into improving the photophysical and chemical properties of microbial phytochrome-based dyes in the last decade (Marx, 2014). Fluorescence quantum yields have increased, molecular weight has decreased, and excitation wavelengths are both extended farther to the red (above 700 nm) while also being available in multiple colors (Figure 1). The use of these tools promises to extend fluorescence imaging to live animals. Further development in all of these areas will bring phytofluors into quotidian use.

The family of phytochromes shares a conserved photosensory protein core consisting of a PAS (Per/Arndt/Sim) domain, a GAF (GMP phospho-diesterase/adenyl cyclase/FhlA) domain and a PHY (phytochrome) domain. While full-length phytochromes are required for biological activity, fluorescence protein development is concentrated to PAS and GAF domains, which together form a chromophore-binding domain (CBD). Wild-type BphPs are dimers, but the strength of the dimerization interface varies among phytochromes (Takala et al., 2015). To increase BphP utility as a fluorophore, residues in this GAF dimer interface have been rationally mutated to create a monomer (Bhattacharya et al., 2014; Yu et al., 2014).

This initial monomeric CBD from *Deinococcus radiodurans* (DrCBD_mon_) has a low fluorescence quantum yield (0.029 ± 0.001; Auldridge et al., 2012; Bhattacharya et al., 2014). In order to rationally improve this yield, one can imagine engineering the protein to affect changes in the kinetics of the competing processes that take place in the excited state, in particular internal conversion or isomerization in the BV C15 = C16 double bond leading to the first relatively stable photoproduct (Lumi-R). Much attention has been paid in particular to the Y263F and D207H substitutions, in large part because of the critical roles these positions play in the normal photocycle (Sineshchekov et al., 2014). Recently it has been shown that the H207 residue is not required for enhanced fluorescence of Infrared Fluorescent Protein (IFP)1.4 (Shu et al., 2009; Bhattacharya et al., 2014) and does not markedly increase fluorescence in Wisconsin Infrared Phytofluor (WiPhy = DrCBD_mon_-Y263F/D207H) (Auldridge et al., 2012). Indeed in IFP2.0, this position is a Thr (Yu et al., 2014). The side chains introduced by these mutations change the hydrogen-bonding network of the binding pocket (Toh et al., 2010, 2011a,b; Zienicke et al., 2011, 2013; Auldridge et al., 2012; Bhattacharya et al., 2014; Yu et al., 2014).

Our motivation in this study has been to explore the effects of a nonpolar substitution of residue 207, which is in closest proximity to the four nitrogen atoms of BV and the ordered pyrrole water found interacting with three of them. We chose to substitute Leu because it is the nonpolar side chain whose structure most closely mimics that of the native Asp. The size of Leu should prevent adventitious binding of Protoporphyrin IXα (PPIXα), which interacts covalently with H207-carrying variants (Fischer and Lagarias, 2004; Wagner et al., 2008; Lehtivuori et al., 2013; Burgie et al., 2014). The evidence for PPIXα binding includes the fact that fluorescence spectroscopy of the D207A apoprotein assembled with BV detected two fluorescent species, one matching the absorption and emission spectra of incorporated PPIXα, and a second matching those for BV (Fischer and Lagarias, 2004; Lehtivuori et al., 2013).

In this paper we engineered two DrCBD_mon_ variants containing D207L; DrCBD_mon_-D207L itself and DrCBD_mon_-Y263F/D207L (WiPhy2). We present a detailed comparative analysis of the spectroscopic properties of these two variants, as well as the three-dimensional structure of WiPhy2. This structural and spectroscopic study improves the integrated understanding of the fluorescence properties of BphPs.

## Materials and methods

### Cloning

Unless otherwise indicated, all reagents and solvents were obtained from commercial suppliers and used without further purification. Novel constructs were made by QuickChange mutagenesis (Stratagene, La Jolla, CA) using an existing pET21a plasmid encoding DrCBD_mon_ with N-terminal T7 and C-terminal hexahistidine tags (Auldridge et al., 2012). The following primers were used to introduce the appropriate mutations: D207L: 5′TTTCCCGCGTCGCTCATTCCGGCGCAGGCC3′; 5′TGCGCCGGAATGAGCGACGCGGGAAAACGG3′ and Y263F: 5′CATGCACATGCAGTTCCTGCGGAACA3′; 5′CATGTTCCGCAGGAACTGCATGTGCA3′. Correct sequences of clones were verified using DNA sequencing at the University of Wisconsin-Madison Biotechnology Center.

### Protein purification

Constructs encoding DrCBD_mon_ variants were transformed into BL21 (DE3) expression cells and grown at 37°C in LB-amp (0.1 mg/ml ampicillin). At OD_600_ 0.5, cells were induced with isopropyl—β-D-1-thiogalactopyranoside at 28°C. Cells were harvested after 4 h by centrifugation at 5000 × g for 30 min, resuspended in lysis buffer (25 mM Tris buffer, pH 8.0, 50 mM NaCl, 5 mM imidazole), and lysed by sonication. After clarification by centrifugation at 40,000 × g for 30 min, the supernatant was incubated with a final concentration of 0.16 mM BV (Frontier Scientific Inc., Logan, UT) in the dark overnight. Proteins were affinity-purified under green light using nickel-nitrilotriacetic acid resin (Qiagen, Valencia, CA). Further purification was performed using hydrophobic interaction on a phenyl-Sepharose column (GE Healthcare) to separate apo- and holophytochrome. Ammonium sulfate was added to the protein at a final concentration of 0.35 M prior to loading. All buffers were filtered and degassed before use. Purified samples were dialyzed overnight against a 200-fold excess volume of (30 mM Tris·HCl, pH 8.0). Finally samples were concentrated to 20 mg/ml, flash-vitrified, and stored at −80°C. Unless otherwise indicated, the samples were kept in the dark before and during the experiments.

### Structure determination by X-ray crystallography

Purified WiPhy2 protein was crystallized by hanging drop vapor diffusion with drops containing a 1:1 mixture of protein and reservoir solution (20% PEG400 and 0.1 M phosphate citrate buffer at pH 4.0). The crystal used for data collection at LS-CAT was soaked for 5 min in a cryoprotectant of 20% glycerol in mother liquor before vitrification.

Data were collected at the Advanced Photon Source, beamline LSCAT 21-ID-D at a wavelength of 0.9787 Å on a Mar 300 CCD detector. The data were integrated and scaled using HKL2000 (Table 1).

**Table 1:** **WiPhy2 X-ray data collection and structure determination statistics**.

	**Rotating anode (PDB ID 4ZRR)**	**LS-CAT ID-D (PDB ID 4Z1W)**
**DATA COLLECTION**		
Wavelength, Å	1.5418	0.9785
Resolution^*^, Å	47.60–1.50 (1.60–1.50)	23.8–1.30 (1.35–1.30)
Space Group	C2	C2
Unit Cell [a, b, c (Å), β (°)]	94.8, 55.1, 69.8, 91.9	94.4, 53.3, 65.7, 90.9
No. Unique Reflections	57,159	80,665
No. Unique Reflections Obs.	54,864	77,741
Completeness, %	96.0 (91.2)	96.5 (94.3)
Redundancy	2.9 (2.0)	7.7 (7.7)
<I/σI>	14.69 (4.83)	33.2 (4.0)
Wilson B value, Å^2^	10.8	10.5
R†_sym_, %	5.0 (17.9)	5.2 (35.3)
**REFINEMENT**		
Resolution, Å	25.0–1.50 (1.54–1.51)	23.22–1.30 (1.33–1.30)
R_work_/R_free_, ^‡^ %	16.0/19.5 (18.8/27.5)	14.4/16.0 (15.1/17.5)
Rms deviations Bonds, Å	0.007	0.006
Rms deviations Angles, °	1.39	1.31
**Ramachandran Statistics**		
Allowed	98.6	98.0
Generously allowed	1.4	2.0
**No. Atoms**		
Protein	2547	2551
Ligand	86	86
Water	288	231
<**B factor**>**, Å^2^**		
Protein	18.5	16.5
Ligand	9.1	7.8
Water	28.4	26.1

* *The highest resolution bin is indicated in parentheses*.

† *R_sym_ = ΣΣj|Ij - < I>|ΣIj, where Ij is the intensity measurement for reflection j and < I> is the mean intensity for multiply recorded reflections*.

‡ *R_work_/R_free_ = Σ||F_obs_|- |F_calc_||/|F_obs_|, where the working and free R factors are calculated by using the working and free reflection sets, respectively. For the R_free_, 5% of the total reflections were held aside throughout refinement*.

The structure was solved in two stages. First, an initial data set collected on a Bruker Microstar rotating anode/R6000 Proteum CCD detector setup from a crystal from the same crystallization experiment was phased by molecular replacement using 4O8G as a model, and refined. This structure was used to phase the higher resolution data set by molecular replacement using Phaser (McCoy et al., 2007). Those reflections assigned to the R_free_ bin were kept consistent.

**Figure 2:**
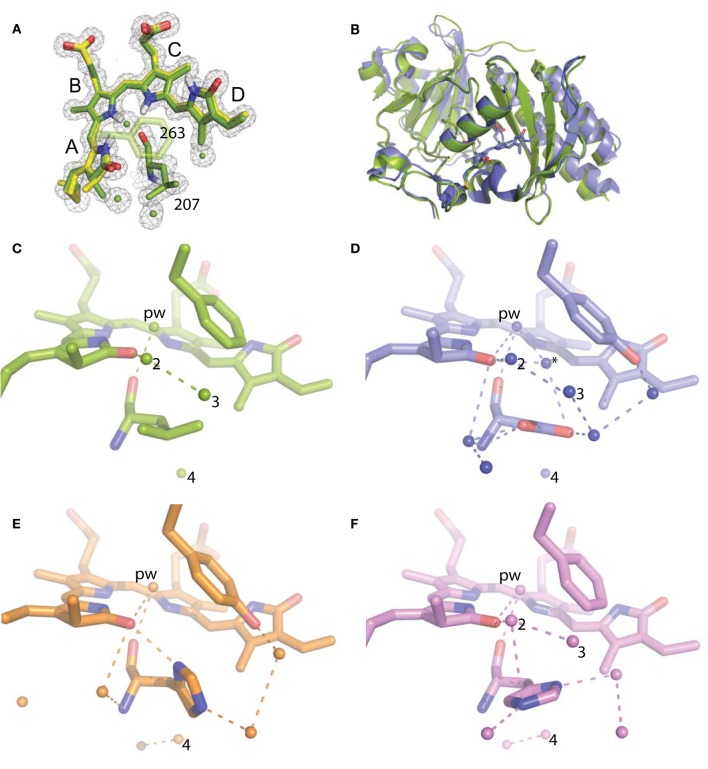
**Structural analysis of WiPhy2 and its water network**. **(A)** BV A-ring methyl in both conformations (green and yellow) and adjacent waters in the WiPhy2 structure. Mesh corresponds to the 2_m_F_o_-DF_c_ electron density map contoured at 1.0 σ and displayed within a radius of 1.6 Å from the chromophore, the waters, or L207. **(B)** Overall structural alignment between DrCBD_mon_ (PDB ID: 4IJG, blue) and WiPhy2 (green). **(C)** WiPhy2 H-bonding network loss in comparison to **(D)** the native sequence monomer (PDB ID: 4IJG), **(E)** the D207H variant (PDB ID: 3S7O, orange), and **(F)** the subsequent addition of the Y263 to create WiPhy (PDB ID: 3S7Q, violet). All waters within 5 Å of any atom within residue 207 are shown as spheres, and H-bonds of up to 3.8 Å are shown between any of the atoms in this set plus the –OH group of BV A-ring or Y263.

For refinement, BV was linked to the Cys24 sulfur with a link entry in the input pdb. To create a library file for the ligand, BV was energy minimized using the Sybyl®-X Suite (Certara) employing the Tripos (Clark et al., 1989) Force Field after correct assignment of the atom types. The restraints for the two enantiomers of the chromophore (designated LBV and LBW) were generated using the Phenix (Adams et al., 2010) routine Elbow without energy minimization. Refinement and model building were carried out in iterative cycles using Refmac5.8.0107 and Coot V0.8.1 (Emsley et al., 2010; Murshudov et al., 2011). The BV chromophore is found with the A-ring C2 methyl occupying both up and down positions, consistent with previously published structures of DrCBD (Figure 2A).

### Spectroscopic measurements

All measurements were carried out at room temperature in complete darkness. Absorption wavelength scans in 1 nm steps from 250 to 850 nm were performed on a Beckman Coulter DU640B and Perkin Elmer LAMBDA 850 spectrophotometers. Sample illumination was as described previously (Auldridge et al., 2012). Briefly, samples were illuminated 15 min with red light or kept in the dark prior to each measurement. The 700 nm light was provided by a Fostec ACE light source fitted with a 700 ± 5 nm interference filter (Andover Corp., Salem, NH). The light source-to-sample distance was adjusted so that irradiances of 150 μmol m^−2^ s^−1^ were used for 700 nm light.

The samples used to record steady-state and time-resolved fluorescence were diluted in (30 mM Tris·HCl, pH 8.0) so that the absorption was sufficiently low (OD_700_ close to 0.1) to prevent an inner filter effect. Fluorescence spectra were measured on a Tecan Infinite M1000 Monochromator-based plate reader with a bandwidth of 5 nm. Emission scans were run in Greiner FLUOTRAC 200 96-well flat-bottom black microplates. The excitation wavelength was 630 nm. The excitation density was kept low to avoid photoconversion of the samples; its absence was confirmed by the identity of absorbance spectra immediately before and after the fluorescence experiments. The fluorescence quantum yields of DrCBD_mon_-D207L and WiPhy2 were determined relative to two reference fluorophores with known quantum yields (Eaton, 1988). Cy5-N-hydroxysuccinimidyl ester (Φ_Cy5_ = 0.27; Lumiprobe) dissolved in phosphate-buffered saline (PBS) and Nile Blue perchlorate (Φ_NileBlue_ = 0.27; Sigma Aldrich) in acidic ethanol [0.5% (v/v) 0.1 M HCl in ethanol] were used as fluorescence quantum yield standards (Sens and Drexhage, 1981; Mujumdar et al., 1993). For pH titration experiments, the protein solution was diluted 50-fold into the appropriate buffer (pH 4-7, 30 mM citrate-phosphate buffer; pH 7-9, 30 mM Tris-HCl; and pH 9 and 10, 30 mM glycine). pH values of aqueous solutions were measured using a standard laboratory pH meter (Fisher Scientific™) calibrated prior to experiments using biotechnology grade standard buffer solutions (pH 4, 7, and 10, Amresco). For the photostability test for WiPhy2 the sample was continuously irradiated (696 ± 5 nm) within the Varian Cary Eclipse spectrophotometer. Power was 2.3 mW, which corresponds to a photon flux of 160 μmol m^−2^s^−1^. Fluorescence intensity was measured at 719 nm every 5 min.

The excitation-emission matrix (EEM) of DrCBD_mon_-D207L and WiPhy2 were recorded on a Varian Cary Eclipse fluorescence spectrophotometer. The EEM fluorescence spectrum was obtained by concatenating emission spectra measured from 630 to 850 nm by using excitation wavelengths of 550-770 nm (5 nm intervals) with 0.1 s integration time and a 5 nm slit widths. The Raman scattering peaks in the EEM spectrum were corrected with a described method (Zepp et al., 2004). The sample resided in a vertically mounted glass capillary with an inner diameter close to 1.1 mm (VITREX, micro-haematocrit) with OD_700_ of about 0.1/mm. To avoid excessive sample degradation, the sample solution (volume 400 μL) was cycled using a peristaltic pump (Ismatec, Reglo Digital) at a flow rate of 0.1 mL min^−1^ through a glass reservoir, the capillary, and connecting Teflon tubing (1 mm inner diameter). A far-red laser diode (750 ± 5 nm, 3 mW, Leading-Tech Laser Co.) was used to transform the sample to the Pr state by constantly illuminating the sample through the Teflon tubing.

Fluorescence decays of the samples in the sub-nanosecond and nanosecond time scales were measured using a time-correlated single photon counting (TCSPC) system consisting of a HydraHarp 400 controller and a PDL 800-B driver (PicoQuant GmBH). The excitation wavelength was 660 nm from a pulsed diode laser head LDH-P-C-660. The repetition rate of the excitation pulses was set to 40 MHz in all measurements, and the output power of the laser was 0.98 mW for 660 nm excitation. The Jobin Yvon monochromator was used to detect the emission at 720 nm with a single photon avalanche photodiode (SPAD, MPD-1CTC). The time resolution was approximately 70 ps [full width at half-maximum of the instrument response function (IRF)]. The data were fitted with monoexponential functions to obtain fluorescence lifetimes (Lehtivuori et al., 2013). In addition to the fluorescence decay components, a fast rise component of about 20 ps was needed to obtain satisfactory fits at early time points.

## Results

### Structural properties

To gain insight into the fluorescent nature of WiPhy2, a 1.3 Å resolution crystal structure was obtained (Table 1). There were no significant changes to the overall structure of WiPhy2 compared to DrCBD_mon_ (RMSD 0.82 Å over all 296 shared Cα atoms including mobile loop regions; Figure 2B). The BV chromophore is well-ordered with no evidence of a break in electron density for the cysteine connection to the A-ring (Figure 2A).

The most obvious result from this new structure is the confirmation of our hypothesis: waters are less abundant around the L207 side chain than has been seen in other high-resolution structures containing either Asp or His at this position. For D207L, within 5 Å of any Leu atom there are only four waters, including the pyrrole water with strong interactions to BV nitrogen atoms in A-, B-, and C-rings (Figure 2C). The closest of these waters to any Leu side chain atom is 3.8 Å. All four water positions are conserved in the water network of DrCBD_mon_ (PDB ID: 4IJG, Figure 2D; Bhattacharya et al., 2014). The second and third form a path from the pyrrole water to the solvent, whereas the fourth is located under the residue 207 side chain and forms a H-bond with Y176. Of course, there are no H-bonds from any of these waters to the L207 side chain in WiPhy2.

This paucity of solvent molecules can be strongly contrasted with the native sequence found in the monomer structure, which holds nine waters (Figure 2D). These waters permit an extensive H-bonding network of 16 different pair wise interactions of 3.8 Å or less between any two atoms in the set containing all atoms in residue 207, the –OH of Y263, the –OH of the A-ring, and these waters.

The introduction of His at position 207 (PDB ID: 3S7O; Auldridge et al., 2012) diminishes this H-bonding network somewhat, with seven waters and nine remaining H-bonds (Figure 2E). For WiPhy (PDB ID: 3S7Q; (Auldridge et al., 2012)), which also has the polar His at position 207 but introduces the nonpolar Phe263, there remain eight waters in the 5 Å cutoff window from the 207 side chain and 9 H-bonds (Figure 2F). Thus, we can conclude that the identity of the residue at position 207 and not at position 263 has the greatest effect on this water network near the “mouth” leading from BV to the solvent. Among the four structures compared here, it is notable that only in DrCBD_mon_ which is known to weakly photoconvert (Auldridge et al., 2012) is there a second water located between the chromophore and this outlet (Figure 2D, asterisk).

### Spectroscopic properties

**Figure 3:**
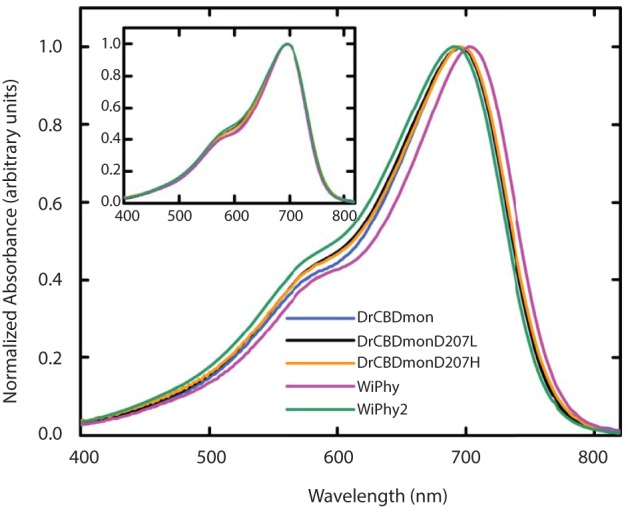
**UV-Vis absorption spectra of the five DrCBD_mon_ variants in the Pr state immediately after sample thawing**. All absorption spectra were normalized at their maxima. (Inset) Spectra are aligned at their absorbance maxima for better visualization of shoulder heights.

In the dark state (Figure 3), both of the D207L constructs show absorption spectra in the characteristic phytochrome region between 600 and 800 nm similar to other monomeric DrCBD_mon_ variants (Auldridge et al., 2012; Bhattacharya et al., 2014; Takala et al., 2015). The D207L constructs have absorption maxima at 696 (WiPhy2) and 697 (DrCBD_mon_D207L), respectively, with a pronounced shoulder at 650 nm. The slight shift (−2 nm relative to DrCBD_mon_) in the maximum absorption wavelength is a hypsochromic shift caused by the decreased hydrogen-bond network around BV compared to DrCBD_mon_ and WiPhy. The amplitude of the shoulder at 650 nm (Figure 3, inset) is higher in the case of the proteins containing Leu at position 207 compared to previously analyzed derivatives of DrCBD_mon_.

**Figure 4:**
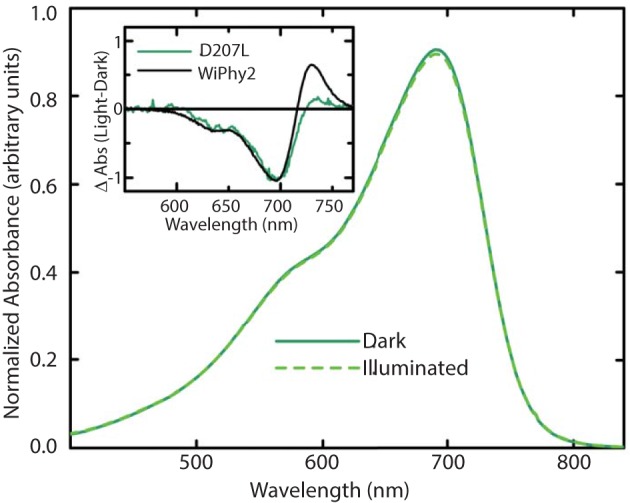
**Steady-state absorption spectra of purified WiPhy2 measured immediately upon thawing (dark) and after 15 min of 700 nm light illumination (illuminated)**. Inset shows absorption difference spectra of DrCBD_mon_D207L and WiPhy2 (red irradiated spectrum has been subtracted from dark spectrum) absorption difference spectra were normalized at 698 nm.

The photoconversion potentials for both D207L variants were also tested (Figure 4). After illumination with red light, the Q band at 696 nm decreases (Figure 4, inset) in each. While native BphP and DrCBD_mon_ as well as other studied variants show an increase in absorbance at 750 nm upon illumination (Auldridge et al., 2012), neither D207L sample has this behavior. Instead, in the two D207L-containing samples, there is a noticeable increase in absorbance in the photoproduct at 730 nm (Figure 4, inset). This difference implies an incomplete or a different type of photocycle than other DrCBD_mon_ variants.

**Figure 5:**
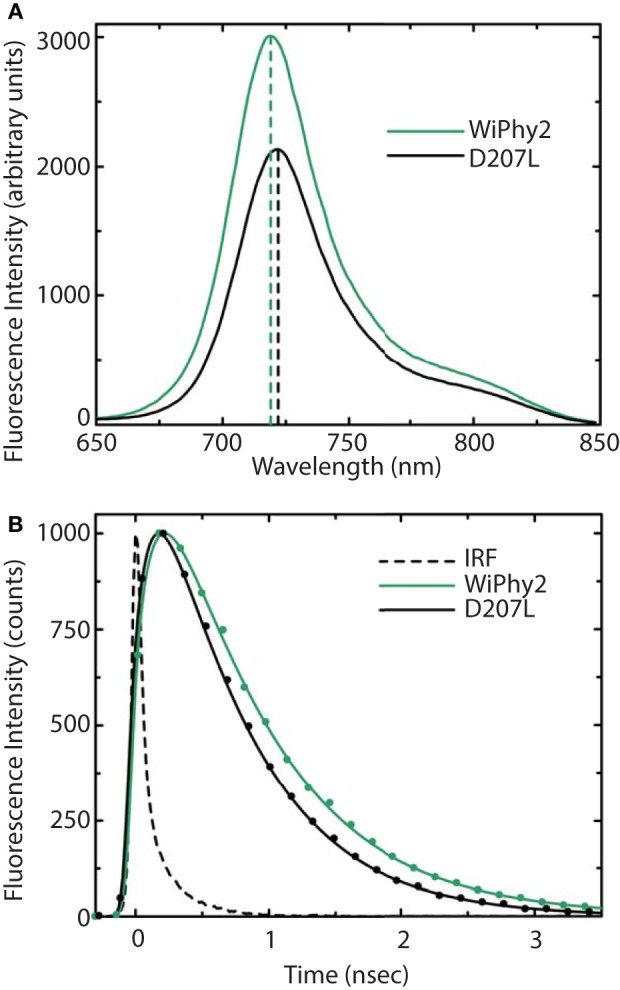
**(A)** Steady-state emission spectra and **(B)** emission decays of purified DrCBD_mon_-D207L and WiPhy2 variants. For steady-state measurements, samples were excited at 630 nm light and intensities were corrected for the number of absorbed photons. Emission decay samples were excited at 660 nm and monitored at 720 nm. IRF is the instrument response function. Solid lines show the multiexponential fit of the data.

The fluorescence spectra of the D207L samples, when excited at the Q-band of BV at 630 nm, are presented in Figure 5A. The observed fluorescence emission originates from the BV chromophore, with the same spectral shape in DrCBD_mon_D207L and WiPhy2, in keeping with their matching absorption spectra (Figure 3). The maxima of the emission spectra are located at 722 and 719 nm for DrCBD_mon_D207L and WiPhy2, respectively. Their fluorescence quantum yields were determined to be 0.070 ± 0.005 (DrCBD_mon_D207L) and 0.087 ± 0.005 (WiPhy2). The quantum yield of WiPhy2 is thus 24% higher than that of DrCBD_mon_D207L.

A similar trend was observed for the time-resolved lifetime measurements (Figure 5B). Using excitation wavelength of 660 nm and monitoring wavelength of 720 nm, the excitation decay properties of BV molecules in the binding pocket can be studied. The excited state decay can be described by monoexponential components, with time constants of 650 ± 30 ps for DrCBD_mon_D207L and 780 ± 30 ps for WiPhy2 (parameters summarized in Table 2).

**Table 2:** **Quantum yield measurements and fluorescence lifetimes**.

**Protein variant**	**Abs max (nm)**.	**Em max(nm)**.	**Φ (%)**	**Lifetime (ps)**	**Binding PPIXα**
DrCBD_mon_-D207L	697	722	7.0 ± 0.5	650 ± 30 ps	ND
WiPhy2(DrCBD_mon_-Y263F/D207L	696	719	8.7 ± 0.5	780 ± 30 ps	No

**Figure 6:**
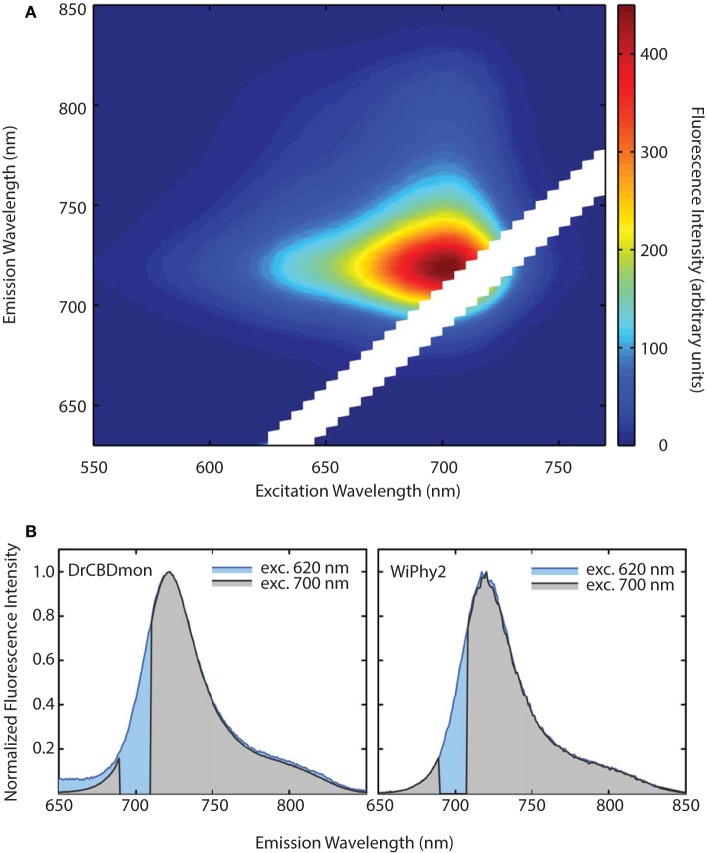
**(A)** Excitation-Emission Matrix (EEM) for WiPhy2 represents fluorescence as a function of both excitation and emission wavelengths. Fluorescence intensities are corrected with the number of absorbed photons. **(B)** Two extracted excitation wavelengths (620 nm and 700 nm) are compared for DrCBD_mon_ (left) and WiPhy2 (right).

The emission-excitation matrix (EEM) spectra for both D207L variants were measured to study with finer detail how fluorescence properties vary with excitation wavelength. We sought to determine whether WiPhy2 binds PPIXα present naturally in the cells it is expressed in. Given that the shape of the fluorescence spectra remains the same in every excitation wavelength (Figures 6A,B), in contrast, for example, to DrCBD_mon_, we conclude that the fluorescence emission from WiPhy2 originates only from the BV chromophore. The composite EEM reveals the change in fluorescence intensity as a function of wavelength and shows a single maximum at ex = 696, em = 719 nm (Figure 6A).

**Figure 7:**
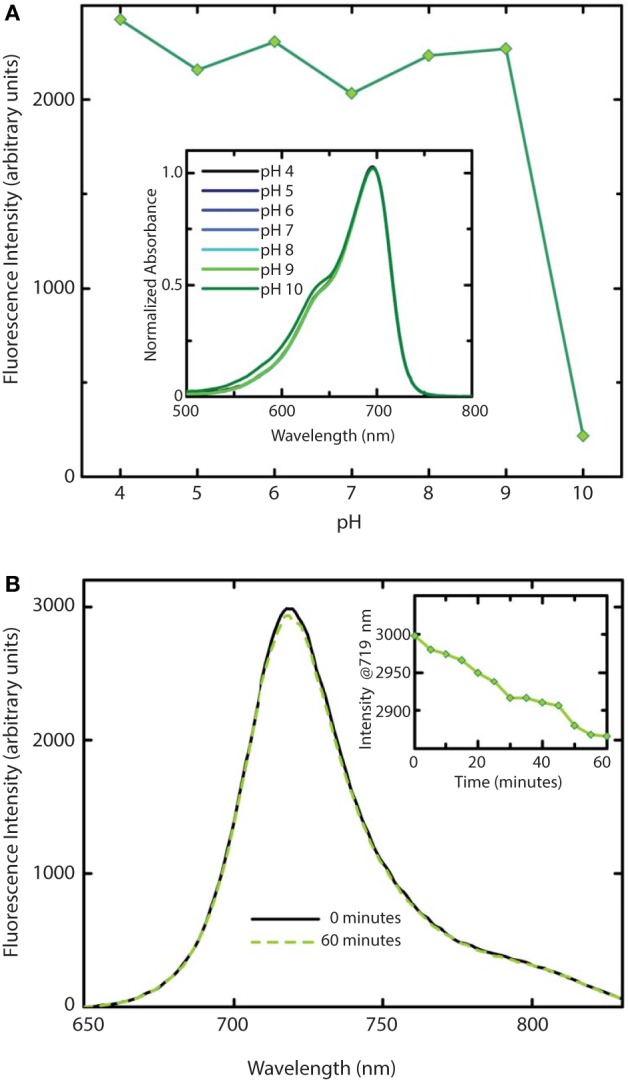
**Stability of WiPhy2 Fluorescence vs. pH and illumination time**. **(A)** Dependency of WiPhy2 absorption (inset) and fluorescence on pH. **(B)** Steady-state emission spectra of WiPhy2 before and after a 1-h photostability test. Inset shows fluorescence intensity at peak over time.

We further investigated the effect of pH on absorption and fluorescence of WiPhy2 (Figure 7A). The fluorescence intensity of sample displayed linear responses to pH values in the range from 4 to 9. The sample was non-fluorescent with pH > 9. Correspondingly, the absorption spectra of WiPhy2 was unaffected by changes in pH ranging from 4.0 to 9.0 (Figure 7A, inset). Drop-off in fluorescence at low pH has been noted with some of the previously published fluorescent variants with His in the 207th position (Filonov et al., 2011) likely due to changes in the protonation state of His (pKa 6.1). This challenge is removed in the case of L207.

Photostability of the WiPhy2 variant was also tested. Samples were excited continuously at their optimal excitation wavelength (696 nm) and fluorescence intensity was measured at 719 nm every 5 min. Fluorescence dropped by only 2% after 60 min (Figure 7B).

## Discussion

Bacteriophytochromes are characterized by structural and spectroscopic variability. Intermediates in their light-driven forward and backward reactions have been trapped at low temperature and spectrally characterized (Eilfeld and Rüdiger, 1985). The first, formed from the excited state bilin molecule on a timescale of ps-ns, is the Lumi-R state. In BphPs, Lumi-R has a ground-state bleach at 700 nm and induced absorption at 730 nm (Toh et al., 2011b). None of the full-length DrBphP Asp207 substitutions stably photoconvert to Pfr upon photoexcitation with red light, although some do reach the Meta-R state, with a steady-state absorption peak between 740 and 750 nm (Borucki et al., 2005; Wagner et al., 2008). By reduction of polarity near the chromophore of WiPhy, we endeavored to limit photoconversion and excited state proton transfer and thus improve fluorescence yield. This goal was informed by the fact that for full-length dimeric DrBph-D207L there is essentially no steady state photoconversion even after extended irradiation (Wagner et al., 2008), and by the fact that two phytofluors in the iRFP series carry D207L substitutions among others.

Here we show that DrCBD_mon_D207L and WiPhy2 respond to red light with miniscule decreases in absorption at 696 nm and induced absorption at 730 nm (Figure 4, inset). Logically, there is also no subsequent appearance of absorbance at 750 nm to indicate the Meta-R or Pfr photoproducts as seen in the parent DrCBD_mon_. Formation of the Meta-R state must require the polar hydrogen-bonding network that is set up by the chromophore, charged residue at position 207, and associated waters (Figure 2). Thus, our study reinforces the major role of Asp207 in the photocycle, and demonstrates that Leu at this position leads to negligible Lumi-R photoproduct yield. Since only steady-state measurements were carried out, we are unable to draw conclusions about a particular reaction scheme for the excited state Pr to the Lumi-R-state. We demonstrate that WiPhy2 has a fluorescence quantum yield of nearly 9% and an excited state lifetime of 780 ps, both ~20% higher than WiPhy (Figure 5), representing the effect of the Leu on the excited state decay of BV in the binding pocket.

The excited state lifetimes for both fluorescent L207 DrCBD_mon_ variants (650 and 780 ps) are longer than for DrCBDmon (620 ps; Bhattacharya et al., 2014) or *Rhodopseudomonas palustris* BphP3 (362 ps; Toh et al., 2011b). Although WiPhy2 lifetime was increased compared to DrCBD_mon_, cyanobacterial Cph1 variant lifetimes have been reported as long as 1.8 ns and IFP_rev_ has a lifetime of 815 ps at room temperature (Miller et al., 2006; Bhattacharya et al., 2014; Kim et al., 2014a). One key reason for variation in lifetimes, apart from the slightly different bilin in Cph1, is immobilization of the D-ring by H-bonding and/or hydrophobic packing (Toh et al., 2011b; Bhattacharya et al., 2014).

We now show that a second mechanism for achieving a longer decay lifetime is to decrease the number of waters near the chromophore, which in turn curtails the hydrogen-bonding network among BV, polar side chains and coordinating water molecules. Opportunities for photoconversion or excited state proton transfer are thus lost. DrCBD variants analyzed to date have clear interactions among solvent molecules (Auldridge et al., 2012; Bhattacharya et al., 2014) but in WiPhy2 there are fewer waters than in any other structurally characterized DrCBD_mon_ variant (Figure 2). Thus, the change to Leu might be viewed as a “mutation” of waters away from the chromophore. We note that the crystals from which these data were obtained have the same C2 space group and are grown under similar solvent content and pH, lending validity to the comparisons. Nonetheless, because cryopreservation conditions may affect overall solvent distributions and because moreover for our spectroscopic studies solution water molecules can be expected to exchange rapidly, our conclusion focuses on this trend rather than a particular water constellation. Following the same logic, our conclusions are not affected by the existence of heterogeneity in the ground or excited states (Samma et al., 2010; Song et al., 2011; Kim et al., 2014b). Thus, X-ray crystallography confirmed the spatial observations of spectroscopic studies; removing polar interactions in the vicinity of the chromophore shifts steady state absorption band location, photocycle yields and fluorescence properties.

Previously it has been demonstrated that phytochromes' absorbance shoulder around 650 nm is not due to a second chromophore species (such as PPIXα) but is instead a natural physical consequence of vibronic progressions in the absorption spectrum (Spillane et al., 2009, 2012). PPIXα binding to the CBD has been a noted disadvantage in several described phytofluors. In the case of *Dr*BphP D207H and as first described in cyanobacterial phytochrome variants (Cph1 Y176R in particular) by Lagarias and coworkers, not only the linear chromophore but also cyclized PPIXα is accommodated in the binding pocket (Fischer and Lagarias, 2004), as observed in emission spectra upon excitation at 600–650 nm. Lehtivuori et al. have shown these minor emission bands due to PPIXα at 660 nm in DrCBD-D207H as well as to some extent in DrCBD (Lehtivuori et al., 2013). Burgie et al. obtained the same result in the case of DrCBD and its D207A variant (Burgie et al., 2014). For both of our D207L variants this PPIXα binding disadvantage is alleviated, as seen in our EEM spectra. Indeed, regardless of the excitation maximum, we obtain same emission spectra of only BV (Figure 7).

We have used steady state and time-resolved spectroscopy as well as protein crystallography to test the hypothesis that the introduction of a nonpolar amino acid in place of the native charge in DrCBD would improve its fluorescence properties. Indeed, in WiPhy2 Leu at position 207 raises fluorescence quantum yield and lengthens excited state lifetime, maintaining an illumination wavelength of nearly 700 nm while avoiding PPIXα binding or a narrow pH window for efficacy (Figure 1).

## Funding

The research was supported by the Academy of Finland grant 277194 (HL), University of Jyväskylä (HL), the Fulbright Center in Finland (HL), the National Science Foundation 1518160 (KTF), and the W. H. Peterson Fellowship (SB). Use of the Advanced Photon Source, an Office of Science User Facility operated for the U.S. Department of Energy (DOE) Office of Science by Argonne National Laboratory, was supported by the U.S. DOE under Contract No. DE-AC02-06CH11357. Use of the LS-CAT Sector 21 was supported by the Michigan Economic Development Corporation and the Michigan Technology Tri-Corridor (Grant 085P1000817).

### Conflict of interest statement

The authors declare that the research was conducted in the absence of any commercial or financial relationships that could be construed as a potential conflict of interest.
